# Neck-specific exercise improves impaired interactions between ventral neck muscles in chronic whiplash: A randomized controlled ultrasound study

**DOI:** 10.1038/s41598-018-27685-7

**Published:** 2018-06-25

**Authors:** Gunnel Peterson, David Nilsson, Johan Trygg, Anneli Peolsson

**Affiliations:** 1 Centre for Clinical Research Sörmland, Uppsala University, Eskilstuna, Sweden; 2 Department of Medical and Health Sciences, Division of Physiotherapy, Faculty of Health Sciences, Linköping University, Linköping, Sweden; 3 Computational Life Science Cluster (CLiC), Department of Chemistry, Umeå University, Umeå, Sweden

## Abstract

Chronic pain and disability is common in whiplash-associated disorders (WAD), leading to personal suffering, sick leave, and social cost. The cervical spine is heavily dependent on muscular support and whiplash injury can cause damage to the neck muscles, but diagnostic tools to measure neck muscle impairment and evaluate exercise interventions are lacking. Therefore, the present study investigated ventral neck muscle interactions in 26 individuals with chronic WAD randomized to neck-specific exercise (NSE) or remaining on a waiting list (WL) in 3 months. We performed real-time, non-invasive ultrasound measurements with speckle tracking analysis and calculated the deformation area and deformation rate in three ventral neck muscles. Multivariate statistics were used to analyse interactions between the muscles. After 3 months of NSE, significant improvements were observed in neck muscle interactions and pain intensity in the NSE group compared to the WL group. Thus, this study demonstrates that non-invasive ultrasound can be a diagnostic tool for muscle impairment and used to evaluate exercise interventions in WAD and stands to make a breakthrough for better management in chronic WAD.

## Introduction

The sudden acceleration-deceleration force transmitted to the neck in whiplash injury can lead to persistent neck pain and disability. The annual incidence is 3 to 6% among adolescents and adults^1,2^, and up to 50% of them experience continued symptoms more than 1 year after the accident^3^, which implies an annual increase in individuals suffering from chronic whiplash-associated disorders (WAD). The cervical spine is heavily dependent on muscular support^4,5^. Neck muscles and tendons produce adequate stability via muscle recruitment and the reflex response from the neural system^4^. In particular, the deep ventral neck muscle is essential for stability in the cervical spine^5^. The whiplash injury can cause damage in facet joints, ligaments, muscles, and/or nerves in the neck^6,7^, albeit no damage is visible on X-ray or standard magnetic resonance imaging (MRI). This damage may decrease stability in the spine and lead to compensatory reorganization in neck muscle activation. Morphological changes with fatty infiltrate have been demonstrated in the ventral muscles^8^. In the dorsal neck muscles, fatty infiltrate occurs within 4 weeks to 3 months after the injury^9^ and is associated with poor functional recovery. Electromyography has shown increased activity in the superficial sternocleidomastoid (SCM) muscle in WAD^10^ and delayed activity in the deep muscles longus capitis (Lcap) and longus colli (Lco) in chronic neck pain^11^. Increased pain after repeated arm lifts has been reported in WAD^12^ and may indicate that the deep neck muscles do not maintain cervical spine stability during arm lifting tasks and/or that the superficial neck muscles are overused.

Diagnostic tools for measuring deep ventral neck muscle function during muscle activation are lacking^11,13,14^. An electromyography method using a nasopharyngeal electrode has been utilized to measure the deep ventral neck muscle in chronic neck pain^11,14^, but it cannot distinguish between Lcap and Lco or detect alterations in muscles below the C3 level, is invasive, and cannot be used in clinical practice. Furthermore, muscle functional MRI (mfMRI) can only evaluate post-exercise muscle function and the method is relatively expensive^13^.

Recently, ultrasound investigations using speckle tracking analysis were used to simultaneously evaluate both deep and superficial ventral neck muscles^15,16^. This non-invasive method can measure muscle deformation, which is the elongation or shortening of the muscle (i.e. displacement of skeletal muscles), during real-time movement, and deformation rate, which is how fast the deformation occurs. For healthy individuals, there was an individual muscle pattern balance of low deformation and deformation rates or high deformation and deformation rates in SCM/Lco, SCM/Lcap and Lcap/Lco^15^. This correlation was altered in individuals with WAD, which may indicate diminished cooperation between superficial and deep neck muscles^15,16^. It can be difficult to analyse and understand many highly correlated variables from relatively few unique individuals. However, it is possible to identify and describe patterns^17^ using multivariate statistics, and this method has been used in clinical diagnostic research^18^. Principal component analysis and projections to latent structures^17^ can be used to identify patterns in multivariate datasets^19^. This method was used to investigate neck muscle function in chronic WAD and healthy controls^16^. A new model of neck muscle function was developed for the ventral neck muscles^16^, indicating a reorganization of muscle function in chronic WAD. Although neck-specific exercise (NSE) may be beneficial by restoring neck muscle function, investigations of the response of neck muscle impairments to exercise are scarce. A small pilot study showed that exercises for ventral and dorsal neck muscles in chronic WAD decreased fatty infiltration in the multifidus muscle^20^ and cranio-cervical flexion exercises improved ventral neck muscle impairments in general neck pain^21^. It is important to identify responders to different exercise programmes based on biological diagnosis (impairments) and psychological risk factors^22^. Exercises are recommended for WAD, but no consensus is available regarding the best treatment for chronic WAD^23,24^ due to conflicting results^25–28^. An NSE programme aimed at increasing neck muscle endurance reported improvements in patients’ self-reported pain and disability^29–31^, but whether NSEs restore impaired interactions between ventral neck muscles is not known.

The aims of the present study were to compare the interaction between three ventral neck muscles (SCM, Lcap, and Lco) after 3 months of NSEs versus remaining on a waiting list (WL) without exercises, investigate whether 3 months of NSE improved ventral neck muscle function compared to the model for healthy individuals developed in an earlier study^16^, and investigate the relationship between ventral neck muscle interactions, neck pain, neck muscle endurance, and self-reported neck disability.

## Results

### Differences between the NSE and WL groups

**Figure 1:**
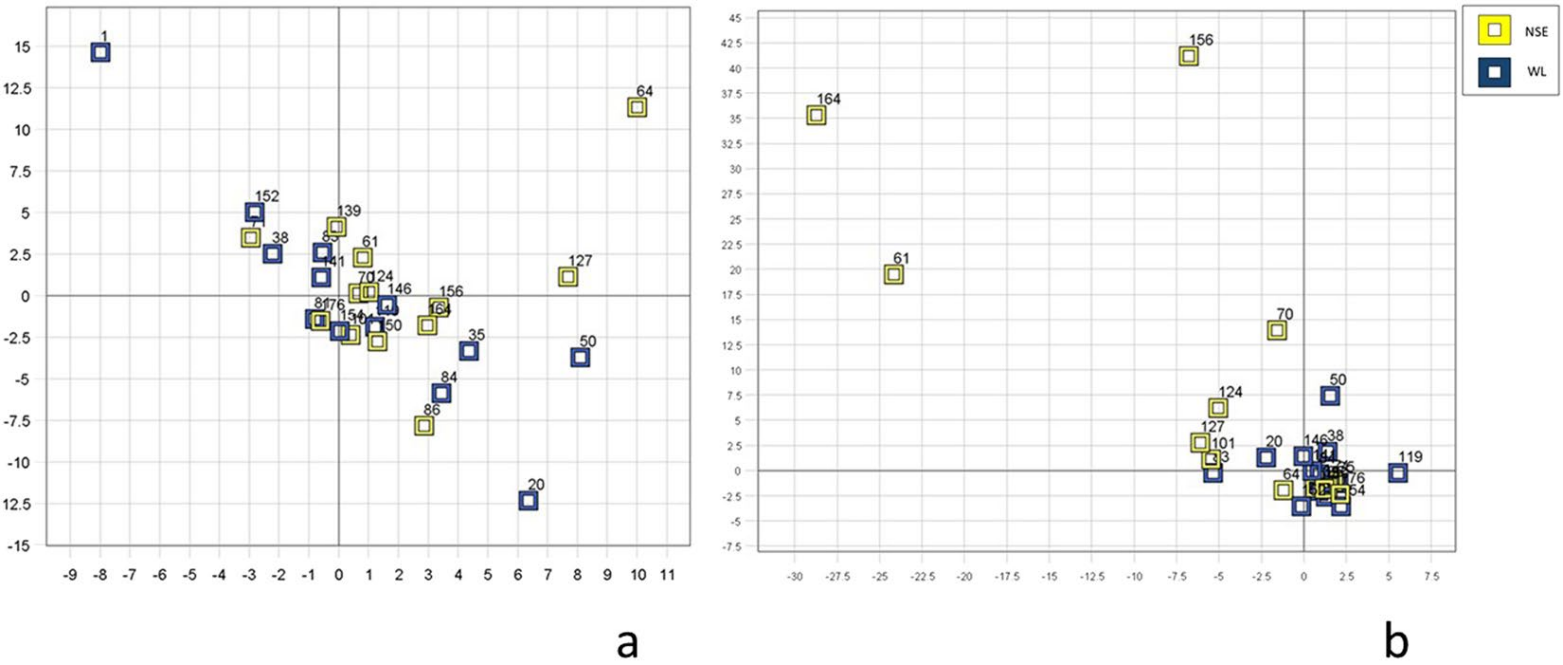
Principal component analysis score plot. (**a**) No significant differences were found between the neck-specific exercise (NSE) group (n = 13, yellow squares) and the waiting list (WL) group (n = 13, blue squares) at baseline (p = 0.24). (**b**) Significant differences were found between the two groups at 3 months (p < 0.03). In the NSE group (yellow squares), seven individuals were positioned more to the left in the model, indicating a ventral neck muscle pattern similar to that of the neck muscle interaction model of healthy controls developed in an earlier study^16^.

The between-group results demonstrated no differences in ventral neck muscles interactions (PCA analyses; p = 0.78, effect size = 0.03, Fig. 1a), pain, neck muscle endurance, or neck disability between the NSE and WL groups at baseline (p > 0.29, Table 1). However, significant between-group differences were found at the 3-month follow-up for neck muscle interactions (p < 0.03, effect size = 0.38, Fig. 1b). The NSE group improved significantly over time (p < 0.03), but not the WL group (p = 0.92). In addition, pain intensity decreased at 3 months in the NSE group (p < 0.05), but no other significant between-group differences in neck disability or neck muscle endurance were observed (p > 0.14). Within group analyses showed that the NSE group significantly improved from baseline to 3 months regarding neck disability, pain intensity, and neck muscle endurance (p < 0.05), but the WL group did not (p > 0.29, Table 1).

**Table 1:** Comparison between the neck-specific exercise (NSE) and waiting list (WL) groups at baseline and 3 months.

Outcome measurement	NSE (n = 13)			p NSE	ES	WL (n = 13)			p WL	ES	p change between groups	ES
	Baseline	3 months	Change score	over time		Baseline	3 months	Change score	over time			
NDI^a^	30 (23–39)	22 (15–28)	−6 (−18–3)	<0.02	0.49	28 (23–35)	28 (21–34)	0 (−6–3)	0.76	0.15	0.14	0.32
Pain before test^b^	26 (16–45)	9 (4–27)	−13 (−39–2)	<0.04	0.41	23 (9–47)	34 (6–52)	4 (−11–20)	0.42	0.08	<0.03	0.44
Pain after test^c^	28 (13–45)	13 (5–22)	−8 (−33–3)	<0.02	0.46	21 (9–60)	35 (8–54)	2 (−6–14)	0.67	0.13	<0.05	0.39
NME ventral^d^	20 (11–45)	36 (15–55)	4 (0–33)	0.05	0.38	13 (7–23)	18 (7–40)	3 (−3–10)	0.34	0.18	0.29	0.21
NME dorsal^e^	31 (23–107)	71(36–198)	21 (−3–113)	<0.04	0.41	29 (7–135)	35 (10–85)	4 (−30–29)	0.75	0.06	0.19	0.26

Median and inter-quartile range.

NSE; Neck-specific exercise group, WL; Waiting list group, ES; Effect Size.

^a^NDI; Neck Disability Index Score (0–100%), higher scores representing higher disability, median (range).

^b^Neck pain before test; pain intensity measured before the ultrasound test at Visual Analogue Scale (VAS) 0–100 mm, higher rating representing higher pain intensity.

^c^Neck pain after test; pain intensity measured after the ultrasound test at Visual Analogue Scale (VAS) 0–100 mm, higher rating representing higher pain intensity.

^d^NME ventral; ventral neck muscle endurance measured in seconds.

^e^NME dorsal; dorsal neck muscle endurance measured in seconds.

### Improvement in ventral neck muscle interaction at 3 months in the NSE and WL group compared to healthy controls

In the NSE group, (n = 13) seven individuals (three men and four women) demonstrated improved ventral neck muscle interactions (p < 0.01), with neck muscle interactions similar to healthy individuals after 3 months of exercise (Fig. 1b). One individual in the WL group demonstrated improved ventral neck muscle interactions at the 3-month follow-up (p > 0.28).

### Ventral neck muscle interaction, pain intensity, neck disability, and neck muscle endurance

The pain intensity, measured immediately after the ultrasound test, negatively correlated with ventral neck muscle interactions at 3 months for the non-improved individuals in the NSE group (r = 0.90, p < 0.02). Despite decreased pain, ventral neck muscle interactions were not significantly improved for six individuals in the NSE group compared to the model for healthy controls^16^. No other correlation was found between ventral neck muscle interaction, pain, neck disability (r = 0.58, p > 0.28) or neck muscle endurance (r = 0.71, p > 0.11). However, the seven improved in ventral neck muscle interaction showed higher ventral [improved; 50 (IQR 36–74), non-improved; 17 (IQR 13–35)] and dorsal [improved; 170 (IQR 43–307), non-improved; 55 (IQR 28–83)] neck muscle endurance at follow-up compared to non-improved, but the differences between group was non-significant (p > 0.11, effect size = 0.44).

## Discussion

In the present study, 3 months of neck-specific exercises improved neck muscle interactions (i.e., the interactions between deep and superficial ventral neck muscles) in individuals with persistent WAD, defined as persistent pain and neck disability 6 months to 3 years following whiplash injury. Ultrasound speckle tracking analysis showed that neck muscle impairment can be normalized despite chronic disability and pain after whiplash injury.

In the NSE group, seven individuals presented a similar muscle interaction pattern as healthy controls after 3 months NSE. NSEs were initially targeted to the deep neck muscles and focused on improving motor control in flexor (longus capitis and longus colli), extensor (multifidus, semispinalis cervicis), and rotator (rotatores) muscles. Additionally, NSE aims to improve muscle endurance in all muscle layers including the superficial ones (extensor: semispinalis capitis, extensor and rotator: trapezius, flexor and rotator: sternocleidomastoid). NSE have demonstrated clinically important improvements in neck disability and pain for roughly 50% of those who attended more than half of the sessions during 12 weeks of training^29,30^. However, for the remaining 50%, the improvements were small or non-existent. Despite decades of research on neck pain and disability in WAD, relatively little is known about the impact of exercise interventions on neck muscle impairment. The reason for the conflicting results or for only some participants in randomized controlled studies to demonstrate a benefit of exercise intervention may be the inability to address the best management of individuals’ impairment in chronic WAD.

In the present study, a postural test of the ventral neck muscles was evaluated. A previous study^16^ showed an important difference between WAD and healthy controls in this postural test. The interactions in healthy controls, including more shortening of the deep muscles, can reflect an active contraction in the deep neck muscles and may therefore be important for the motor control of the cervical spine^16^. Moreover, the elongation of the deep neck muscles seen in WAD may reflect difficulties in maintaining a stable cervical spine during active arm movements. Six individuals in the NSE group did not improve in ventral neck muscle interaction, despite decreased pain intensity. Pain experience is multifactorial^3^, one of which may be altered neck muscle interactions between different muscle layers. Decreased pain may be due to psychosocial factors such as an active coping style that were not investigated in this study. Some individual with longstanding severe pain may also need a longer time to improve than others. However, individuals with low back pain experience fewer recurrences in the long term after an exercise intervention targeting the deep multifidus muscle^32^. The multifidus muscle recovery was not spontaneous upon reduction of painful symptoms in the control group, and muscle recovery was rapid and more complete in the specific exercise group. The same may occur in persistent WAD, as the recovery of muscle interaction may protect from recurrences in the long term. The results in the present study may also indicate that the interplay between neck muscles is not a valid method for investigating motor control of the cervical spine. However, a procedure involving test sets was employed to validate the methodology and prediction efficiency^16^. The robust validation procedure could predict highly significant differences between WAD and healthy controls in ventral neck muscle interactions^16^, and showed elongation of the deep neck muscles in WAD as well as altered interactions between superficial and deep neck muscles. The results in the present study may therefore indicate that some individuals need other exercise interventions and/or a longer exercise period to improve ventral neck muscle interactions.

Little evidence and conflicting results in highly qualitative randomized controlled trials^26–30^ have raised the question of whether exercise plays any role in the management of chronic WAD. General exercise or an exercise programme with few exercises targeting the deep neck muscles seems to not improve disability and/or neck pain^27,28,33^. Not all individuals with WAD may have altered neck muscle function. Recently, investigation of the interplay between ventral neck muscles^16^ showed that, although most of the individuals with WAD had different muscle interplay than healthy controls, five out of 23 individuals with WAD had similar muscle interplay as the asymptomatic control group.

Exercises in WAD are recommended to be selected based on the assessed impairments^22^; however, few studies have investigated impairments in the deep neck muscle layers before and after exercise interventions in WAD. In a pilot study, O’Leary *et al*.^20^ showed that enhanced fatty infiltrate in the deep multifidus muscle in WAD grade II can be modified after 10 weeks of neck exercises targeting the upper and lower neck flexor and extensor muscles, beginning with isometric, and later isotonic, endurance and strength exercises (50–80% of maximal voluntary contraction). In addition, neck disability decreased after 10 weeks of exercises and the morphological muscle impairments in the deep multifidus muscle could be changed with training. However, no reductions in fatty infiltrate were observed in the deep ventral muscles longus capitis and longus colli, and the study included only five individuals and no control group. Therefore, more studies are warranted to investigate and improve exercise interventions aimed at restoring neck muscle impairments. In general neck pain, neck-specific exercises improved impairments in the deep and superficial neck muscles^21^, but the electromyography method used to measure the deep muscles is invasive, uncomfortable, and not applicable in clinical practice. The stability of the cervical spine is heavily dependent on support from surrounding neck muscles, with 70% of the stability assumed to come from muscles, especially the deepest layers^4^. If exercise interventions are intended to improve both deep and superficial muscles, the ability to diagnose muscle impairments is urgent, especially when exercise interventions in WAD are debated^22,24,34^. The results in the present study are very promising, and ultrasound with speckle-tracking analysis can be used for improved diagnosis and evaluation of neck muscle impairments in WAD, which could lead to improved exercise interventions.

Studies of general neck pain^35,36^ have shown that it is related to neck muscle impairment. Falla *et al*.^36^ showed that higher pain levels are associated with delayed and lower electromyography amplitude in deep ventral muscles at cranio-cervical flexion contraction. Increased coactivation between ventral and dorsal neck muscles also correlated with increased pain^35^. Neck muscle strength (% maximal voluntary contraction) moderately correlated with the current neck pain level at test^37^, a strategy that may protect the spine from further injury and pain^38^. However, correlation between ventral neck muscle endurance and longus colli size were reported in both neck pain patients and healthy controls^39^, but no correlation was found between pain intensity and neck muscle function as muscle size and proprioception. In recent years, studies have shown a more individual response to pain^40–42^. In neck muscles, individual-specific muscle activation has been reported, with increased activation in a given muscle in some individuals and decreased activation in the same muscle in other individuals^42^. In the present study, the results indicate that improvement in the interaction between deep and superficial neck muscles could occur despite pain, and despite decreased pain intensity, the ventral muscle interaction was not improved. However, the study sample was small and the findings need to be confirmed in larger randomized studies.

This study has some limitations. First, the small sample size may have caused the insignificant differences between groups because of a lack of power in NDI and NME, and further sub-group analyses were not possible. Also, the aetiology of chronic WAD with pain persisting from 6 months to 3 years following whiplash injury is heterogeneous, which may have affected the results. However, the study showed significant within group results for the NSE group for neck disability and neck muscle endurance and can be a starting point for further evaluation of neck muscle impairments and exercise interventions in WAD grade II and III. Further studies with larger sample size is required to confirm the results.

In conclusion, ultrasound measurement with speckle-tracking analysis demonstrated that ventral neck muscle impairment in chronic WAD improves with NSE. Individuals in the NSE group exhibited an interplay between deep and superficial ventral neck muscles similar to healthy controls after 3 months of training. The results are promising for further evaluation and improvement of exercise interventions in individuals with persistent pain and disability after whiplash injury.

## Materials and Methods

### Design

Individuals with persistent pain and disability after whiplash injury that occurred 6 to 36 months prior to inclusion were included in this prospective, randomized controlled study (ClinicalTrials.gov NCT01547624, registered March 8, 2012) with blinded outcome assessments.

### Procedure

Individuals were consecutively recruited for ultrasound investigation from a larger ongoing randomized controlled trial^43^. Participants were recruited from primary health care centres, specialist orthopaedic clinics, and hospital outpatient services between February 2011 and May 2012 (recruitment and follow-up). To be eligible for the ultrasound study, individuals had to report right-handedness and right-sided neck pain. Other inclusion criteria were positive manual examination findings corresponding to WAD grade II (neck pain and musculoskeletal signs) or III (neck pain plus neurological signs)^44^; persistent neck pain > 20 mm on a visual analogue scale (VAS) and/or neck disability > 20% measured with the neck disability index (NDI)^45^; age 18–63 years; and ongoing symptoms associated with a whiplash injury that occurred 6 months to 3 years prior to study entry. For exclusion criteria see Table 2.

**Table 2:** Exclusion criteria

*Signs of traumatic brain injury at the time of whiplash injury
*Known or suspected serious pathology
*Previous fracture or luxation in the cervical spine
*Contraindication to exercise
*Neuromuscular disease
*Rheumatological disease
*Previous serious neck pain that warranted more than 1 month of sick leave in the year prior to the whiplash injury
*Severe mental illness
*Current alcohol or drug abuse.
*Insufficient knowledge of the Swedish language (with inability to answer the questionnaires)

Written informed consent was obtained from all participants. The study was approved by the Regional Ethics Review Board, Faculty of Health Science Linköping University (Dnr 2010/188-31) and was conducted according to the Declaration of Helsinki.

**Figure 2:**
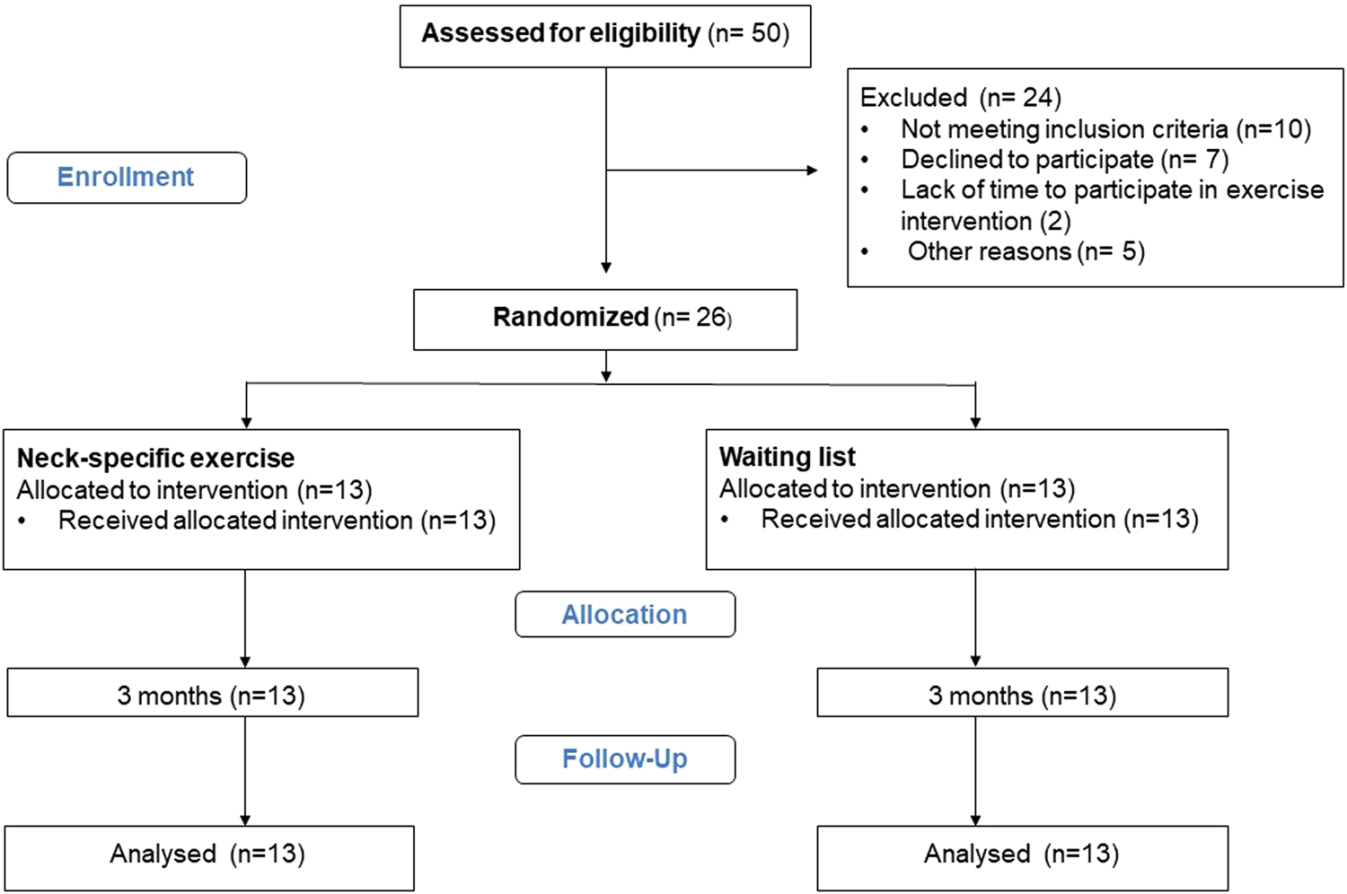
Study flow chart.

Twenty-six individuals were included in the study and randomly assigned (Fig. 2) to NSE (10 women and 3 men; median age 41 years, range 30–48 years) or remaining on a WL (11 women, 2 men; median age 40 years, range 27–47 years) for 3 months (Table 3). As no previous study has compared the interaction between ventral neck muscles after 3 months of NSE versus remaining on WL without exercises, the sample size was arbitrary.

**Table 3:** Participant characteristics.

Characteristic	NSE (N = 13)	WL (N = 13)	p
Gender; (number; female/male)	10/3	11/2	0.99
WAD grade II/III (number)	10/3	6/7	0.23
Age (year)	41 (30–48)	40 (27–47)	0.53
Injury duration^a^	18 (14–26)	20 (11–27)	0.87
BMI^b^	25 (22–30)	23 (20–29)	0.52
Physical activity level^c^	2 (2–3.75)	3 (2–3)	0.89
Neck Disability Index (0–100%)^d^	30 (23–39)	28 (23–35)	0.56
Neck pain now (VAS 0–100)^e^	27 (13–63)	47 (17–61)	0.73
Neck pain worst (VAS 0–100)^f^	68 (46–77)	70 (50–87)	0.51

^a^Months since whiplash injury, median (range).

^b^Body Mass Index (BMI; median (range)).

^c^Activity index of physical activity level last 12 months (1 = inactivity, 2 = low activity, 3 = moderate activity, 4 = high activity).

^d^Neck Disability Index Score (0–100%) 10 items, higher scores representing higher disability, median (range). Minimal disability (0–20%), moderate disability (21–40%), severe disability (41–60%), crippled (61–80%) bed bound (81–100%).

^e^Visual Analogue Scale (VAS), neck pain now 0–100 mm, higher rating representing higher pain intensity, median (range).

^f^Visual Analogue Scale (VAS), average worst neck pain last week 0–100 mm, higher rating representing higher pain intensity, median (range).

An independent researcher not otherwise involved in the study managed the randomization procedure. A computer-generated list randomly allocated the qualified participants. For a detailed description, please see ref.^43^.

### Intervention

The NSE group performed neck-specific exercises twice weekly for 12 weeks supervised by physiotherapists practising in primary health care with additional home exercises. They followed a standardized exercise protocol with flexibility to modify the programme on an individual basis if required. The first visit to the physiotherapist included information concerning anatomical and physiological factors relevant to symptoms following whiplash injury. The exercises initially targeted activation of the deep neck muscles^29^ and the exercises continually progressed within the participant’s symptom tolerance. The exercise intervention was previously described in detail^29,46^. Participants and treating physiotherapists could not be blinded to group allocation because of the nature of the study. For a detailed description and photos, please see the Academic Archive online: http://urn.kb.se/resolve?urn=urn:nbn:se:liu:diva-113865.

The WL group were offered NSE after the 3 months waiting list period. No one in the WL group remain untreated.

### Ultrasound measurements

**Figure 3:**
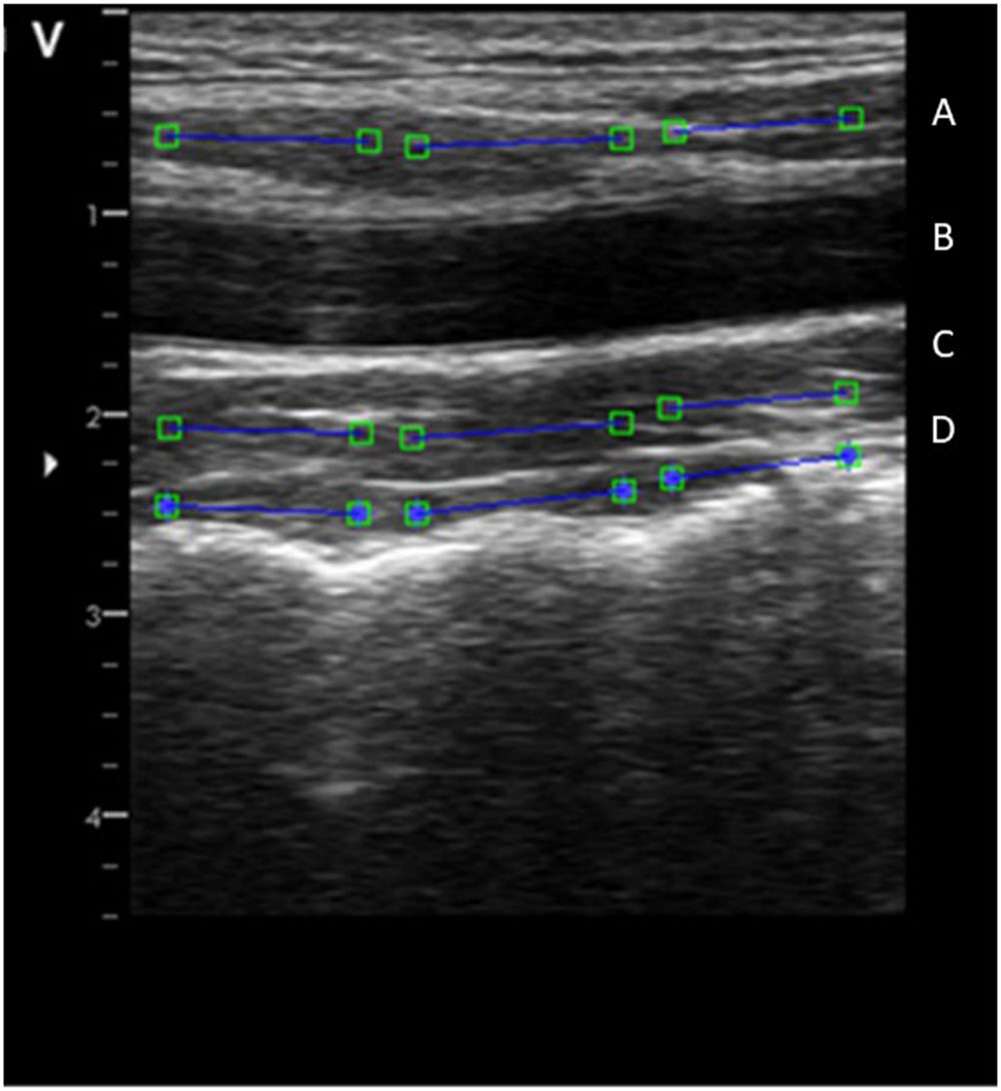
The ultrasound image shows the three range of interest (ROI) manually placed in the first frame of the video sequence of each muscle in longitudinal projection. The superficial sternocleidomastoid (**A**) shown at the top, followed by the common carotid artery (**B**), longus capitis (**C**) and longus colli (**D**). The ROI measuring the deformation (elongation and shortening) and the deformation rate (how fast the shortening and elongation occurs). Each ROI are indicated as a blue line with a square at each end.

**Figure 4:**
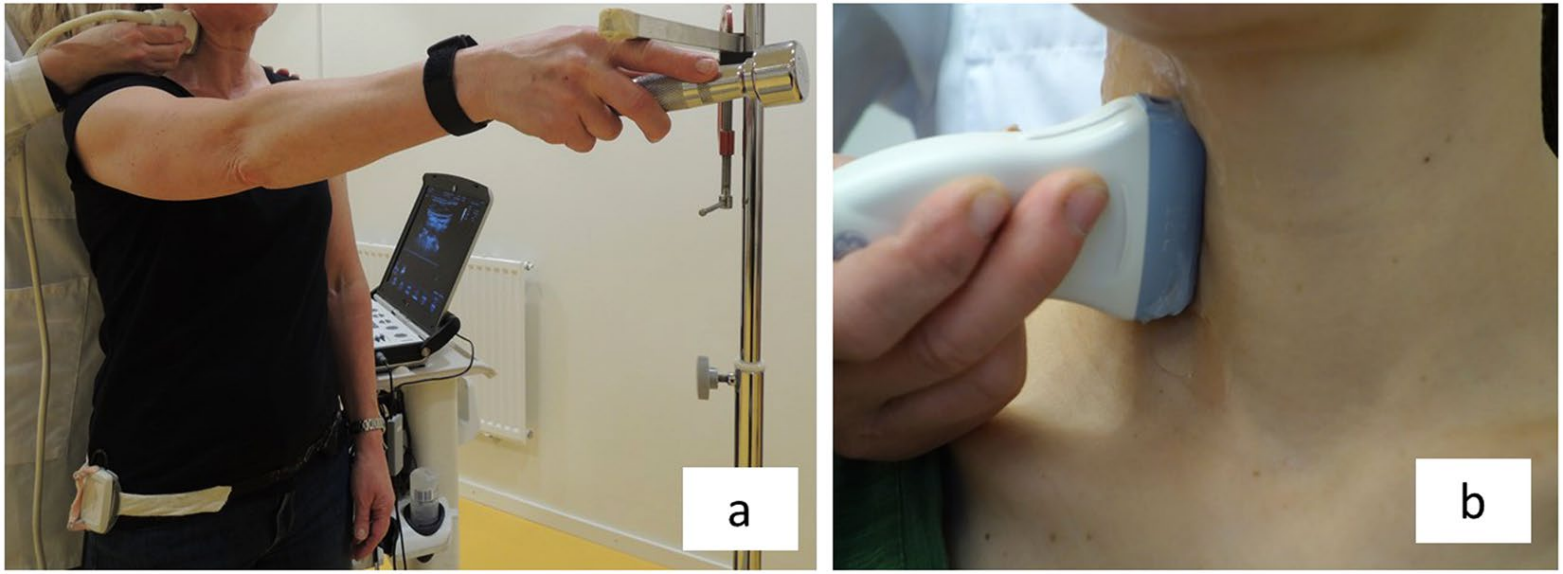
Ultrasound measurement. (**a**) Ultrasound imaging of ventral neck muscles during arm elevation. The individual held a 0.5 kg (women) or 1 kg (men) weight in the right hand and raised their arm to 90 degrees. An adjustable horizontal bar was fixed with the index finger touching the bar and customized contact switches attached to the individual’s hip and wrist. The contact switches allowed synchronization between the ultrasonography data and arm elevation. The segmental level was verified with a transverse ultrasound projection of the bifurcation of the carotid artery, commonly observed at the C4 level. (**b**) The transducer was then rotated 90° and orientated longitudinally to the ventral neck muscles.

The ventral neck muscles were recorded using a B-mode, 2-D ultrasound Vivid-i scanner (GE Healthcare, Horten, Norway) and a 12 MHz linear transducer (38 mm) at 235 frame/s. The SCM, Lcap, and Lco muscles were recorded (Fig. 3) during 10 repetitive arm elevations. Real-time ultrasound images (“video” sequences) were obtained during the first and tenth arm elevations. The transducer was positioned at the C4 level on the right side of the neck. The segmental level was verified by projection of the bifurcation of the carotid artery commonly observed at the C4 level. The transducer was then rotated from a transverse to a longitudinal position, which allowed optimal imaging of the SCM, Lcap, and Lco muscles. All ultrasound measurements were obtained in this longitudinal position on the right side of the neck (Fig. 4a,b, written consent was obtained to publish the photo, Supplementary file).

### Speckle tracking

Ultrasound produces sound waves with frequencies higher than the limit of human hearing (the limit of human hearing is <20 000 hertz, and ultrasound for medical imaging generates frequencies up to 100 times higher than the human hearing range at 2 to 20 MHz), and echoes returning off the muscles can be recorded to visualize grey-scale images (B-mode). The ultrasonic probe produces the waves and the returning echoes serve as acoustic markers, forming a unique speckle pattern. This speckle pattern can be followed frame-by-frame through the ultrasound images to obtain measurements of muscle deformation, i.e. displacement of skeletal muscles (elongation or shortening) and the deformation rate (i.e., the rate at which the deformation occurs) using post-process speckle tracking analysis.

Three region of interest (ROI) was manually placed in the first frame of the video sequence of each muscle (Fig. 3). The unique speckle pattern of the respective ROI was tracked based on an algorithm developed by Kanade Lukas-Tomasi^47,48^ and further enhanced by Farron *et al*.^49^. This stable mathematical model^47–49^ with at least 80% agreement between frames were sufficient to find the patterns of speckles and follow the changes in muscle deformation. When the muscle speckle pattern changes length, the tracked ROI also changes in length. The frame to frame displacement was obtained with a least squares fit assuming a linear strain model. The displacement of all points within the ROI was summed to gain a cumulative sum from all frames in the movie that provided quantitative information on muscle behaviour during the arm elevation.

Muscle deformation, defined as a change in ROI length and considered as displacement of muscles (elongation or shortening), was calculated as the percentage change (% deformation) from the original length. The muscle deformation rate was calculated as the amount of deformation per time unit (% deformation/s). Three ROIs (each 10 × 3.3 mm) were positioned longitudinal to the muscle fibres in each of the three muscles (SCM, Lcap, Lco). Lopata *et al*.^50^ reported that the magnitude of muscle deformation measured with speckle tracking is positively related to other measurements used to investigate muscle deformation (e.g., force measurements and progressive electrical stimulation). The speckle tracking analysis method has been shown to have excellent test-retest reliability (two-way random absolute agreement single measure intraclass correlation coefficient [ICC] 0.71–0.97)^51^.

**Figure 5:**
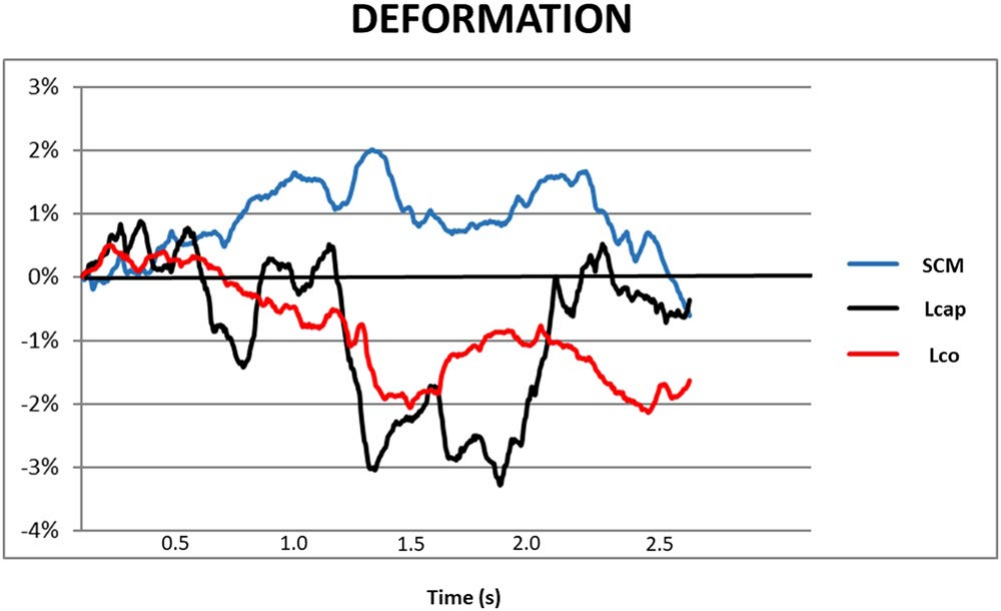
The deformation area in three ventral neck muscles during one arm elevation in a single participant. The y-axis is the deformation in percentage (%) and the x-axis is the the time in seconds. Each line indicates the changes in the ROI (deformation %) of one muscle during one arm elevation: m. sternocleidomastoid, SCM (blue line); m. longus capitis, Lcap (black line); and m. longus colli, Lco (red line). The negative values (area below zero) are muscle shortening and the positive values (area above zero) muscle elongation. The sum of the negative and positive areas is the total area and denotes the total muscle deformation. The muscle switches from shortening to elongation when the line crosses zero, and vice versa.

To estimate muscle deformation, the areas on the deformation curves were calculated (Fig. 5). The trapezoidal rule was used for the area calculation (Equation 1) where A is the area, t is time between samples and yn is the current ROI position at sample point n. To handle intersections with the 0% line, the equation was modified. Linear interpolation was used to estimate additional sample points with adjusted t-values at intersections with the 0% line. Thus, the area under and the area above the 0% line could be separated.

1 $${\rm{A}}={\rm{t}}/{\rm{2}}\,\mbox{''}({\rm{y1}}+{\rm{2y2}}+{\rm{2y3}}+\mathrm{..}+{\rm{2yn}}-{\rm{2}}+{\rm{2yn}}-{\rm{1}}+{\rm{yn}})$$

### Test procedure

The individual held a 0.5 kg (women) or 1 kg (men) weigh in the right hand and the arm raised to 90 degrees, with the index finger touching an adjustable horizontal bar (Fig. 4a). Each individual performed 10 arm elevations. Ultrasound images were taken of the first and tenth arm elevations. Customized contact switches were attached on the right hip and the right wrist and the contact signals recorded in the ultrasound machine. This allowed data synchronization and gave information on the start and stop of arm movements. A metronome was set to 40 beats per minute to keep a steady pace during the examination (each test took approximately 3 seconds).

### Other measurements

Neck pain intensity was measured immediately before and after the ultrasound test and assessed using a VAS (0–100 mm scale, 0 = no pain, 100 = worst imaginable pain)^52^. Neck disability was measured using the NDI, which consists of 10 items expressed as a percentage (total possible score 100%), with higher scores indicating greater disability^45^. Ventral neck muscle endurance was measured in seconds with the patient supine with the head and cervical spine in a neutral position, arms positioned alongside the body, and legs straight. Dorsal neck muscle endurance was measured with the patient prone and the head initially supported on the examination table, arms alongside the body, and legs straight. A load of 2 kg for women or 4 kg for men was applied to the head and the individuals instructed to lift their head just above the examination table. The neck muscle endurance test was previously described in detail^53^.

### Data analysis

Ultrasonography data were post-process analysed by speckle tracking implemented with a program designed in-house for Matlab^54^. To evaluate muscle deformation, the areas on the deformation curves were calculated for the first and tenth arm elevations (Fig. 5). The ultrasound video images were coded during the post-process analyses. Therefore, the analyser was blinded to the group affiliation.

### Statistical analysis

**Figure 6:**
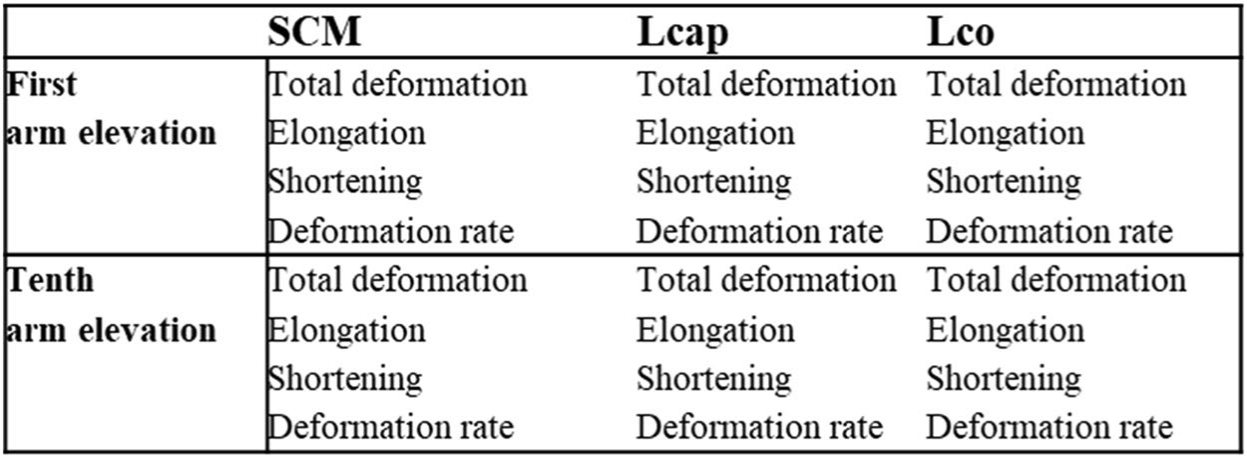
The 24 variables included were the Sternocleidomastoid (SCM), Longus capitis (Lcap) and Longus colli (Lco); the deformation area (% deformation) and deformation rate (% deformation/s) throughout the first and tenth arm elevation; the area of total muscle deformation; the areas of shortening and elongation deformation; [3 muscles (SCM, Lcap, Lco) × 2 conditions (deformation and deformation rate) × 3 variables (total deformation, shortening and elongation = 24].

The variables collected from the study comprised deformation areas and deformation rate from the SCM, Lcap, and Lco muscle layers during the first and tenth arm elevations (Fig. 6). Thus, a total of 24 variables were obtained from the three neck muscles. Two-way interaction terms (quadratic terms included) were calculated for all possible pairs of the 24 variables. Variable mean-centring and scaling to unit variance was applied to the 24 variables prior to calculating the two-way interaction terms. The interaction between two variables, *a* and *b*, can be written as:

2 $${i}_{ab}=\frac{a-{\bar{x}}_{a}}{{s}_{a}}\,^\circ \,\frac{b-{\bar{x}}_{b}}{{s}_{b}}$$

where ${\bar{x}}_{a}$ and ${\bar{x}}_{b}$ are the variable means and *s*_*a*_ and *s*_*b*_ are the standard deviations (Equation 1). Quadratic terms were logarithmically scaled to base 10. The 300 variables, including original variables, quadratic terms, and two-way interactions, were assembled in a data matrix and subjected to multivariate data analysis. The results from the present study were compared to raw data from an earlier study in which a model of ventral neck muscle function was developed for chronic WAD and healthy controls^16^.

Multivariate analysis was performed on the three correlated muscle layers simultaneously using Evince (Evince 2.6, Prediktera AB, Umeå, Sweden). In a previous study^16^, principal component analysis was used to summarize the variation in the data and to create overviews in the form of principal component analysis score scatter plots. The original principal component analysis model from the previous study including 43 individuals was used to predict the individuals assigned to the NSE and WL groups in the current study. For the prediction, the first principal component was used because it had the most pronounced difference between individuals suffering from WAD and healthy controls. Predicted principal component analysis scores for the individuals in the NSE and WL groups were obtained by multiplying each individual *i* with the loading vector of the first principal component (Equation 3).

3 $$tpre{d}_{i}={x}_{i}{p}_{1}$$

The predicted score values for the individuals in the NSE and WL groups were used as inputs for the subsequent statistical analyses. All statistical data analyses were performed in SPSS statistical software, version 24. Because of the small sample size, all data were analysed by non-parametric statistics. Effect sizes were calculated (non-parametric; Z-score divided by the square root of the number of total observations, Equation 4).

4 $$r=\frac{Z}{\sqrt{N}}$$

Between group differences were analysed by the Mann Whitney U test or the chi^2^ test. The Wilcoxon signed-rank test was used for paired two-group analyses over time.

### Data availability

The datasets analysed during the current study are available from the corresponding author on reasonable request.

## Electronic supplementary material

Consort checklist

Protocol
