# Supplementary material for: Neck-specific exercise improves impaired interactions between ventral neck muscles in chronic whiplash: A randomized controlled ultrasound study
Source: Sci Rep. 2018 Jun 25;8:9649. doi: 10.1038/s41598-018-27685-7 (PMC6018626; doi:10.1038/s41598-018-27685-7)
Supplement: Supplementary file 2 — Protocol [file 41598_2018_27685_MOESM2_ESM.docx]

**Neck Muscle Activation in Patients With Long Standing Problems After a Whiplash Trauma Registered With Ultrasound With Speckle Tracking**

**Background**

Today there is evidence for that neck specific exercises reduce neck pain problems in patients with mechanical neck pain (1). However, there is a lack of consensus on how to optimize neck exercises and dosages in neck rehabilitation after whiplash injury. More knowledge is needed to determine which and how muscles are activated in exercises thought to be neck-specific. There is neither enough knowledge of differences in neck muscle activation between patients with long standing neck problems after a whiplash trauma and healthy controls (2-5) nor if the neck muscle function will improve with specific neck exercises (6). One approach would be a real time study of deformation and deformation rate in different layers of neck muscles in patients with residual long-term neck pain and disability after a whiplash trauma.

**Objectives**

The purpose of the present study is to investigate deformation and deformation rate in different layers of dorsal and ventral neck muscles (including upper and middle part of trapezius muscle) during rest and during different exercises; arm flexion until 120 degrees, repeated arm flexion until 90 degrees, low loaded neck extension, low loaded neck muscle endurance test, shoulder elevation.

**Methods**

Forty patients with long standing (more than 6 months but less than 3 years) pain and disability after whiplash injury and 40 controls matched for age and gender will be included for comparisons between patients and healthy in neck muscle activation. The patients will be consecutively recruited for ultrasound investigation from a larger ongoing randomized controlled trial. The patients will also be randomized to A) neck specific exercises in a 3 months period and B) be on a waiting list for 3 months. Measurements will be performed at baseline and at 3 months follow-up.

To be eligible for the ultrasound study, patients have to report right-handedness and right-sided neck pain. Other inclusion criteria is positive manual examination findings corresponding to WAD grade II (neck pain and musculoskeletal signs) or III (neck pain plus neurological signs) (30); persistent neck pain > 20 mm on a visual analogue scale (VAS) and/or neck disability > 20% measured with the neck disability index (NDI) (31); age 18 – 63 years; and ongoing symptoms associated with a whiplash injury that occurred 6 months to 3 years prior to study entry. Exclusion criteria is signs of traumatic brain injury at the time of whiplash injury; known or suspected serious pathology; previous fracture or luxation in the cervical spine; contraindication to exercise; neuromuscular diseases; rheumatologic disease; previous serious neck pain that warranted more than 1 month of sick leave in the year prior to their whiplash injury; severe mental illness; or current alcohol or drug abuse.

*Ultrasound imaging*

To evaluate the neck muscles deformation (elongation and shortening of the muscle) and deformation rate (how fast the deformation occur) we will use a B-mode, 2-D ultrasound Vivid-i scanner (GE Healthcare, Horten, Norway) and hand-held 12-MHz linear array transducer (12 L-RS, footprint 39 mm, GE Healthcare,Horten, Norway) with high frame rate (235 frames/s). Measurements will be made from the ultrasound at the level of the fourth cervical vertebra, which is identified by palpation of the C-4 spinous process.

*Speckle tracking*: Real-time ultrasound imaging of skeletal muscle is able to detect the unique speckle pattern in muscles and can be analyzed post-processing using the speckle

tracking method based on an algorithm developed by Kanade, Lucas and Tomasi (7, 8) and Farron et al. (9). A region of interest (ROI) is manually placed in the recorded muscle

images and make it possible to track the unique speckle pattern frame by frame through the ultrasound video-sequence. Each ROI consisted of a large number of measuring points and is placed in the first frame in the ‘‘video’’ sequence, following the frame to frame deformation

throughout the ultrasound imaging. This provides measurement of muscle deformation) and is calculated as the percentage change in the ROI from the first original frame length of the ROI (expressed as % deformation). The muscle deformation rate is expressed as the

deformation per time unit (% deformation/s).Three rectangular ROIs (each 10 x 3.3 mm) are

manually placed longitudinal to the muscle fibers in each muscle; together, the three ROIs cover 30 mm.

*Intervention*

The neck-specific exercise program is supervised by a physiotherapist twice weekly for 12 weeks, with additional home exercises. The patients is given information concerning anatomical and physiological factors relevant to symptoms after whiplash injury,

including the need for postural awareness. The exercises is initially low load and targeted at the deeper ventral and dorsal neck muscle layers. At weeks 2 to 3, the patients commence exercises designed to improve NME using weighted pulleys and guild boards. These

exercises will be continually progressed within the participant's symptom tolerance. Participants in will be instructed to avoid pain aggravation during exercise.

**Ethical considerations**

Written informed consent will be obtained from all participants. The study is approved by the Regional Ethics Review Board (Dnr 2010/188-31) and conducted according to the Declaration of Helsinki.

**Project management**

The project leader is Anneli Peolsson, Associated Professor in physiotherapy, certified as a specialist in Orthopedics and is international leading in her research field, Linköping University, Sweden.

Main collaborator in the present study include Gunnel Peterson MSc, Phd-student at Linköping University, Sweden and Reg. Physiotherapist (specialist in pain and pain rehabilitation, ultrasonography, behavior science and chronic pain conditions).

Other researcher involved in the project; Michael Peolsson, PhD, medical engineer, specialist in ultrasonography, Linköping University Sweden, and for multivariate analyses Johan Trygg, Professor at Department of Chemistry Umeå University, Sweden and David Nilsson, PhD Senior research engineer at Department of Chemistry, Umeå University, Sweden.

**Timetable**

Data collection will start in February 2011 and is planned to end in May 2012.

**References**

1. Gross AR, Goldsmith C, Hoving JL, Haines T, Peloso P, Aker P, et al. Conservative management of mechanical neck disorders: a systematic review. J Rheumatol. 2007;34(5):1083-102.

2. Sterling M, Jull G, Vicenzino B, Kenardy J, Darnell R. Development of motor system dysfunction following whiplash injury. Pain. 2003;103(1-2):65-73.

3. Elliott JM, O'Leary S, Sterling M, Hendrikz J, Pedler A, Jull G. Magnetic resonance imaging findings of fatty infiltrate in the cervical flexors in chronic whiplash. Spine. 2010;35(9):948-54.

4. Elliott J, Jull G, Noteboom JT, Darnell R, Galloway G, Gibbon WW. Fatty infiltration in the cervical extensor muscles in persistent whiplash-associated disorders: a magnetic resonance imaging analysis. Spine (Phila Pa 1976). 2006;31(22):E847-55.

5. Jull G, Kristjansson E, Dall'Alba P. Impairment in the cervical flexors: a comparison of whiplash and insidious onset neck pain patients. Man Ther. 2004;9(2):89-94.

6. Jull G, Falla D, Treleaven J, Hodges P, Vicenzino B. Retraining cervical joint position sense: the effect of two exercise regimes. J Orthop Res. 2007;25(3):404-12.

7. Lucas BD, Kanade T. An iterative image registration technique with an application to stereo vision. In: Proceedings, 7th International Joint Conference on Artificial Intelligence (LICAI), August 24–28, 1981, Vancouver, BC, Canada; 1981;. p. 674–679.

8. Tomasi C, Kanade T. Detection and tracking of point features. Pittsburgh,

PA: Computer Science Department, Carnegie Mellon University;April 1991.

9. Farron J, Varghese T, Thelen DG. Measurement of tendon strain during muscle twitch contractions using ultrasound elastography. IEEE Trans Ultrason Ferroelectr Freq Control. 2009;56(1):27-35.
